# High-Precision Pose Measurement of Containers on the Transfer Platform of the Dual-Trolley Quayside Container Crane Based on Machine Vision

**DOI:** 10.3390/s25092760

**Published:** 2025-04-27

**Authors:** Jiaqi Wang, Mengjie He, Yujie Zhang, Zhiwei Zhang, Octavian Postolache, Chao Mi

**Affiliations:** 1 Logistics Engineering College, Shanghai Maritime University, Shanghai 201306, China; 202230210297@stu.shmtu.edu.cn (J.W.); yzgeu@iscte-iul.pt (Y.Z.); 2 Institute of Logistics Science and Engineering, Shanghai Maritime University, Shanghai 201306, China; 202230510050@stu.shmtu.edu.cn; 3 School of Technology and Architecture, ISCTE-Instituto Universitário de Lisboa, 1649-026 Lisbon, Portugal; 4 Shanghai SMU Vision Co., Ltd., Shanghai 201306, China; zhangzhiwei@smuvision.com; 5 Instituto de Telecomunicações, ISCTE-Instituto Universitário de Lisboa, 1649-026 Lisbon, Portugal; opostolache@lx.it.pt

**Keywords:** machine vision, dual-trolley quayside container crane, container-transfer platform, high-precision pose measurement, adaptive image enhancement, multi-scale object detection

## Abstract

To address the high-precision measurement requirements for container pose on dual-trolley quayside crane-transfer platforms, this paper proposes a machine vision-based measurement method that resolves the challenges of multi-scale lockhole detection and precision demands caused by complex illumination and perspective deformation in port operational environments. A hardware system comprising fixed cameras and edge computing modules is established, integrated with an adaptive image-enhancement preprocessing algorithm to enhance feature robustness under complex illumination conditions. A multi-scale adaptive frequency object-detection framework is developed based on YOLO11, achieving improved detection accuracy for multi-scale lockhole keypoints in perspective-distortion scenarios (mAP@0.5 reaches 95.1%, 4.7% higher than baseline models) through dynamic balancing of high–low-frequency features and adaptive convolution kernel adjustments. An enhanced EPnP optimization algorithm incorporating lockhole coplanar constraints is proposed, establishing a 2D–3D coordinate transformation model that reduces pose-estimation errors to millimeter level (planar MAE-P = 0.024 m) and sub-angular level (MAE-θ = 0.11°). Experimental results demonstrate that the proposed method outperforms existing solutions in container pose-deviation-detection accuracy, efficiency, and stability, proving to be a feasible measurement approach.

## 1. Introduction

**Figure 1:**
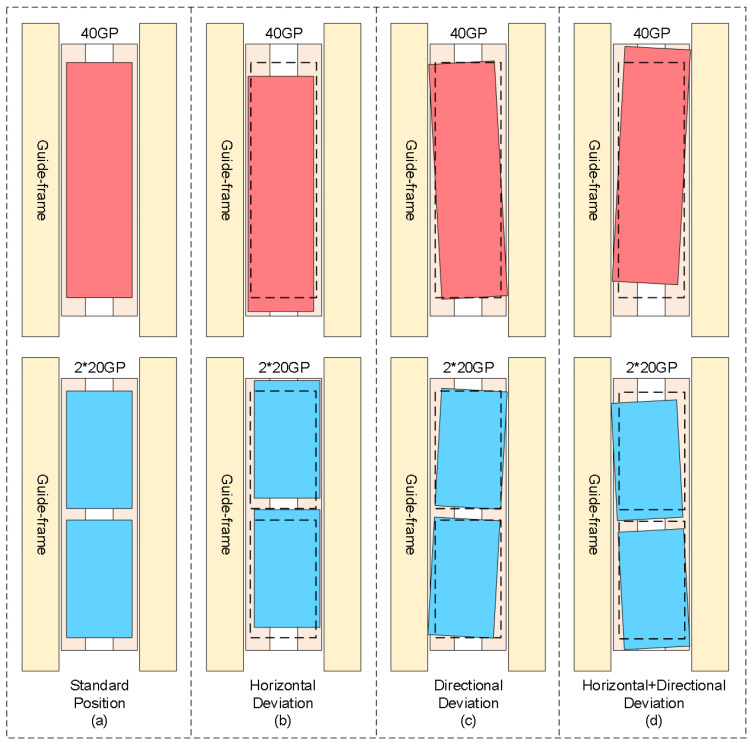
Schematic diagram of horizontal and rotational deviations of containers on transfer platform.

With the rapid development of automated container terminals, dual-trolley quayside container cranes, as core equipment for efficient loading/unloading operations, rely on their critical connecting component—the container-transfer platform—to facilitate container handover between the main trolley and gantry trolley. However, during container-unloading operations, mechanical clearance caused by long-term wear of container guide frames and spreader oscillations frequently result in actual container poses deviating from preset positions on the transfer platform. Figure 1a illustrates standard poses of a 40-foot general purpose container and dual 20-foot general purpose containers on the transfer platform; Figure 1b demonstrates horizontal deviation of containers; Figure 1c depicts rotational deviation; Figure 1d presents combined horizontal–rotational deviations. These deviation patterns necessitate frequent spreader pose adjustments during secondary grasping operations, which critically depend on perception systems’ precise calculation of 3D positions and deflection angles. The accuracy and speed of container pose estimation thus become crucial factors affecting operational efficiency. Enhancing the measurement accuracy and reliability of container positions and orientations on transfer platforms remains a significant research focus. Traditional manual measurement methods suffer from low efficiency and inconsistent precision, while existing vision-based systems face multiple challenges in complex port environments: dynamic lighting conditions (e.g., intense glare, rain/fog interference) causing image feature degradation and target-recognition failures; perspective-induced multi-scale deformations of container top-surface lockholes under fixed camera top-view configurations that hinder traditional image algorithms; and stringent robustness requirements for millimeter-level pose measurement.

This paper introduces a high-precision pose-measurement method for containers on the dual-trolley quayside container crane-transfer platform. The method employs a fixed camera-based pure vision detection approach, utilizing an adaptive image-enhancement preprocessing algorithm to flexibly adapt to complex illumination environments in ports. A multi-scale adaptive frequency object-detection framework based on YOLO11 is developed. The framework combines image characterization at different scales and dynamically adjusts the frequency domain information-processing strategy to more accurately identify and localize targets of different sizes in complex scenes. It can effectively address the issue of excessive scale variations in container lockhole keypoints caused by perspective transformation. Subsequently, the three-dimensional coordinates of container lockhole keypoints are calculated by combining the coplanar characteristics of container top lockholes with an improved EPnP optimization method, achieving millimeter-level horizontal deviation estimation and sub-angle-level deflection angle measurement. The proposed method provides a feasible solution for high-precision container pose measurement on dual-trolley quayside container crane-transfer platforms.

The contributions of this paper are summarized as follows:

1. To address the limitations of low efficiency and unstable accuracy in traditional manual operations for field engineering applications, a machine vision-based high-precision pose-measurement system for containers on the dual-trolley quayside container crane-transfer platform is proposed;
2. To mitigate interference from complex illumination and meteorological interference in port environments on operational site images, an adaptive image-enhancement preprocessing algorithm is designed to strengthen image features;
3. To resolve the challenge of large-scale variations in lockhole keypoints on container tops caused by perspective transformation in operational scenarios, a multi-scale adaptive frequency object-detection framework is developed based on the YOLO11 architecture, enabling robust target recognition and keypoint detection;
4. To overcome the low precision of traditional pose-estimation algorithms, an improved EPnP optimization method is proposed to achieve high-accuracy measurement of 3D container positions and orientations.

## 2. Related Work

Container pose-measurement technology is a core component of automated port operations. Early research primarily relied on manual measurement and sensor-assisted positioning techniques, such as LiDAR-based [1] or inertial navigation-based pose-estimation systems [2]. Although these methods achieve high accuracy, they suffer from high equipment costs, complexity of deployment, and susceptibility to mechanical vibration interference [3]. With the advancement of machine vision, monocular or binocular camera-based visual measurement methods have emerged as research hotspots. Kuo et al. [4] proposed a container damage-detection method based on machine vision, and Ji et al. [5] developed a vision-based truck-lifting accident-detection approach. However, these methods generally face dual constraints of calibration sensitivity and nonlinear computational complexity growth. Notably, monocular vision-based pose estimation has gained significant attention in the last five years due to its deployment flexibility and cost-effectiveness, yet challenges remain in overcoming dynamic illumination disturbances and target scale variations in actual port operations.

The complex optical conditions in port operational scenarios, including strong reflections and rain/fog blurring, pose significant challenges to image feature extraction. Traditional image-enhancement methods (e.g., histogram equalization) exhibit unstable feature performance under non-stationary illumination due to environmental adaptability limitations. Degradation-aware image enhancement is an intelligent enhancement technique that incorporates the analysis of image-degradation factors. Its core idea is to explicitly identify and model the types of degradation in the image (e.g., noise, blur, low resolution, etc.) when improving the image quality (e.g., denoising, deblurring, contrast enhancement, etc.), and adaptively adjust the enhancement strategy based on this information, so as to achieve more accurate and efficient restoration and enhancement. Researchers have proposed various degradation-aware image-enhancement strategies, such as Retinex theory-based dynamic contrast adjustment algorithms [6], which tend to introduce noise under extreme illumination. Recent deep learning-based feature-enhancement methods [7,8,9] demonstrate stronger robustness, exemplified by Li et al.’s [10] attention-guided residual blocks for real-time low-light image enhancement in smart ports. Lin et al. [11] achieved container number recognition with surface contamination and damage through deep learning and low-light enhancement algorithms. Nevertheless, existing methods still struggle with composite interference from large-scale variations and degradation coupling in port scenarios, requiring further optimization of feature fidelity and computational efficiency.

Under fixed camera configurations, container terminal operations frequently encounter drastic target scale variations. Traditional object-detection models like Faster R-CNN [12] face limitations in handling scale diversity due to fixed receptive field designs. YOLO-series algorithm [13] improvements enhance multi-scale detection capabilities through Feature Pyramid Networks [14], yet still suffer from missed detections under extreme scale differences. Zhou et al. [15] integrated SRCNN [16] and Resblock [17] to develop a contour feature-enhancement module, combining DConv [18] for cross-scale feature-enhancement networks. However, this approach incurs substantial computational overhead. Recent studies explore frequency-domain analysis with spatial feature extraction [19], enhancing detail features through high-frequency components while maintaining structural integrity via low-frequency components [20,21], offering new paradigms for multi-scale detection. However, their generalization capability in dynamic port scenarios requires further validation.

The core of 3D pose estimation lies in solving camera extrinsic parameters through 2D–3D point correspondences. Traditional PnP algorithms like DLT and EPnP minimize reprojection errors for pose estimation but tend to fall into local optima under noise interference. The EPnP algorithm reduces computational complexity through virtual control points [22]. Some studies integrate RANSAC mechanisms to enhance noise resistance [23], yet iterative processes compromise real-time performance. Moreover, existing methods rarely exploit practical constraints from real operational environments, leading to redundant degrees of freedom and limited accuracy in pose estimation.

Despite progress in visual detection and pose estimation, significant gaps remain between current technical capabilities and operational requirements for dual-trolley quayside container crane-transfer platforms. Existing image-enhancement methods lack joint modeling capabilities for illumination, meteorological interference, and geometric distortion when addressing dynamic degradation coupling in complex port environments. Traditional convolutional networks struggle with extreme scale variations of container top lockhole keypoints during port operations. The accuracy of the pose estimation remains constrained by detection precision and algorithmic limitations, leaving substantial room for improvement. Current dual-trolley quayside container crane-transfer platform operations still lack effective and reliable solutions for container position and orientation measurement.

## 3. Three-Dimensional Positioning and Pose-Measurement System

### 3.1. Hardware System

**Figure 2:**
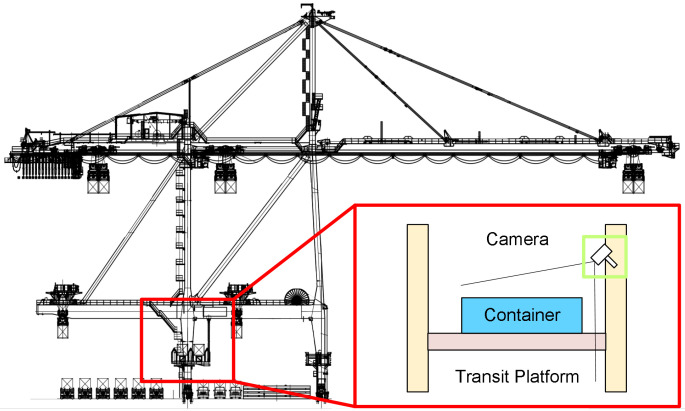
Fixed camera installation location schematic diagram.

This paper proposes a hardware system based on visual measurement for the measurement of three-dimensional positioning and poses of containers on the transfer platform of a dual-trolley quayside container crane. The system consists of fixed cameras and edge computing modules. Fixed visual cameras are installed on the land side column of the quay crane above the transfer platform, as shown in Figure 2. These cameras are tilted downward to capture images, with their field of view fully covering the container-transfer platform to record the operational workflow of container placement.

The edge computing module processes visual data captured from containers on the transfer platform. In actual operations, the fixed cameras continuously capture container images and transmit them to the edge computing module. Through advanced image-processing algorithms and pose-detection algorithms, the module analyzes and processes these images to ultimately obtain accurate container position and attitude information.

### 3.2. Algorithm Design

**Figure 3:**
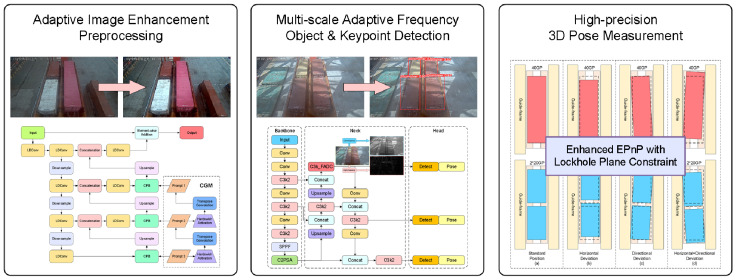
Algorithm flowchart.

The workflow of the 3D positioning and pose-measurement algorithm for transfer platform containers based on visual measurement proposed in this paper is illustrated in Figure 3. First, the video stream captured by fixed cameras is input frame-by-frame as raw images. Image preprocessing is performed using the proposed adaptive image-enhancement preprocessing algorithm, which dynamically adjusts enhancement strategies for complex illumination and meteorological interference in port environments to efficiently enhance image features.

After dynamic image feature enhancement, container recognition/classification, and lockhole keypoint detection are required. To address the large-scale variations caused by perspective transformations in containers and their top lockholes captured by fixed cameras on dual-trolley quayside container cranes, the proposed multiscale adaptive frequency object-detection method is implemented. Based on the YOLO11 framework, this method identifies 20-foot and 40-foot general purpose containers while obtaining two-dimensional image coordinates of lockhole keypoints.

Finally, using the output container-recognition information and 2D image coordinates of lockhole keypoints, the proposed 2D–3D keypoint coordinate-conversion algorithm calculates the three-dimensional coordinates of container lockhole keypoints. These 3D coordinates are then input into the pose analysis algorithm to ultimately determine the container’s offset direction/distance and rotation direction/angle relative to baseline positions on the transfer platform.

#### 3.2.1. Adaptive Enhancement Image Feature Preprocessing Method

**Figure 4:**
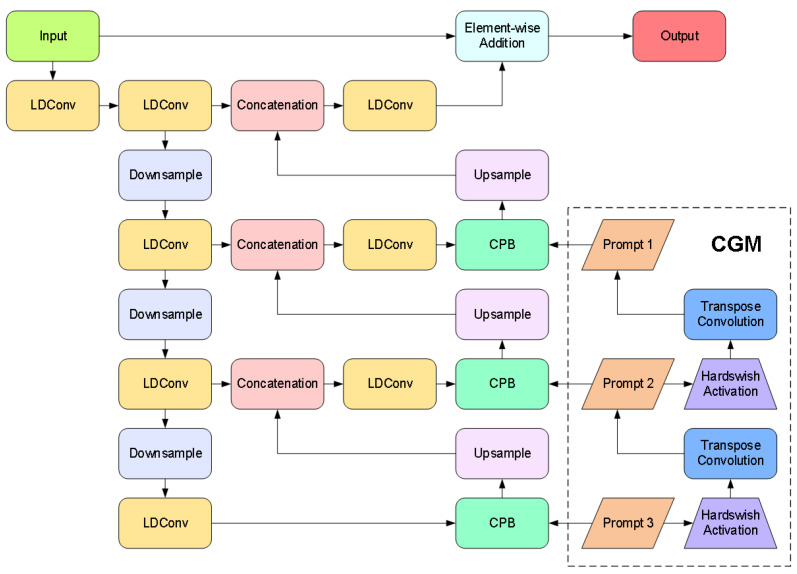
Network structure diagram of adaptive image-enhancement preprocessing method.

To address interference issues caused by high-contrast complex illumination and rain fog blur in complex environments at container terminals that affect container images on transfer platforms, we designed an adaptive enhancement image feature preprocessing algorithm. This algorithm serves as a preprocessing component before image-detection algorithms to mitigate the impacts of complex illumination and meteorological interference. As illustrated in Figure 4, inspired by Zhang et al. [24], we propose a novel method leveraging the characteristics of the Chain-of-Thought Prompt Generation Module (CGM) and Content-Driven Prompt Block (CPB) to enhance port container images under degradation conditions such as complex illumination and environmental challenges.

**Figure 5:**
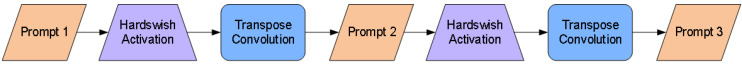
CGM module network architecture diagram.

CGM Module: The Chain-of-Thought prompting mechanism constructs a multilevel degradation-aware semantic encoding framework, as illustrated in Figure 5. Its core employs a transposed convolution sequence to generate resolution-increasing prompts, progressively refining from low-resolution global semantics (Such as rain blur, fog blur, dark light attenuation and other types of degradation categories) to high-resolution local features (Degradation intensity distribution for rain and fog blurring, dark light weakening, etc.). Initial prompts undergo multi-stage upsampling and channel compression, with information filtering achieved through the Hardswish activation function [25]. This establishes inter-level dependencies. The design breaks through the static limitations of traditional independent prompts, guiding the model through chain-of-thought reasoning to parse degradation patterns from coarse to fine. Driven by the training data, CGM automatically learns the features of different degradation types such as rain blur and dark light weakening. Combined with the multi-scale features of the decoder, it significantly enhances adaptive capability for degradation types.

Specifically, the construction of this module primarily consists of following steps. First, an initial prompt is constructed by initializing a tensor of a learnable prompt $P_{3}\in R^{\hat{H}\times \hat{W}\times \hat{C}}$ in the third layer of the decoder, where $\hat{H}\times \hat{W}$ denotes the initial spatial resolution and $\hat{C}$ represents the dimension of the channel. This prompt learns global representations of degradation patterns through backpropagation. Subsequently, multiscale prompt sequences are progressively generated via stacked transposed convolutions—a 3×3 transposed convolution operation upsamples $P_{3}$ to produce the second-layer prompt $P_{2}\in R^{2\hat{H}\times 2\hat{W}\times \hat{C}/2}$, following which the first-layer prompt $P_{1}\in R^{4\hat{H}\times 4\hat{W}\times \hat{C}/4}$ is generated similarly. After each transposed convolution layer, the Hardswish activation function is used to suppress irrelevant information flow, selectively propagating degradation-related characteristics as formulated in Equation (1).

(1) $$P_{i}=\mathrm{Hardswish}\left({\mathrm{TC}}_{3\times 3}\left(P_{i+1}\right)\right),\;i\in \left\{1,2\right\}$$

where ${\mathrm{TC}}_{3\times 3}$ represents the 3×3 transposed convolution operation. The CGM module establishes hierarchical dependency relationships between prompts, enabling coarse-to-fine progressive reasoning of degradation patterns through multiscale feature interactions.

**Figure 6:**
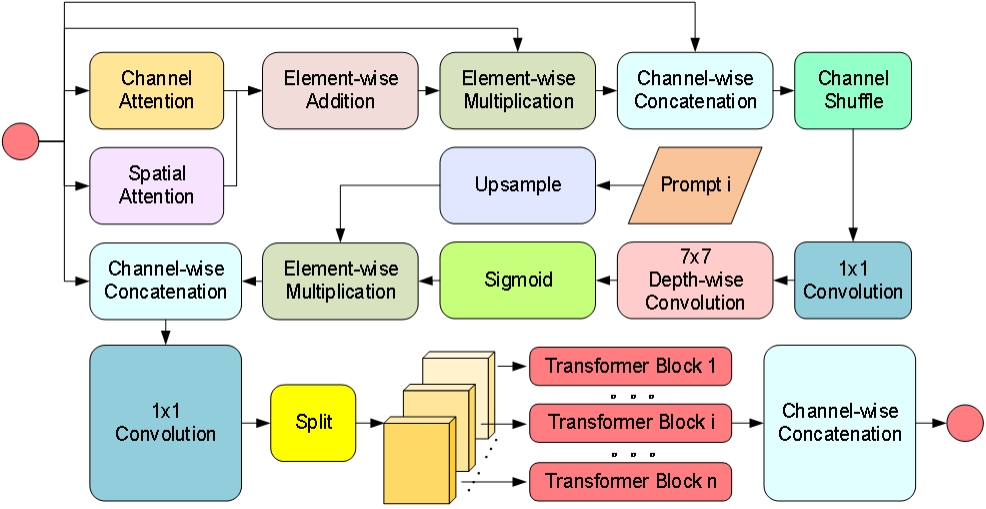
CPB module network structure diagram.

CPB Module: Achieves degradation-aware feature enhancement through dual-path hybrid attention and parallel Transformers as illustrated in Figure 6. First, the channel-spatial attention jointly models feature importance to generate content-sensitive weight distributions. Subsequently, the prompt information undergoes interpolation alignment and concatenation with features. The fused features are then fed into multiple parallel Transformer sub-blocks via a channel-splitting strategy, where cross-channel attention is computed individually and gating mechanisms control information flow. Prompts generated by the CGM dynamically adjust the enhancement strategy by interacting with image features at each layer of the decoder through the CPB module. At the shallow decoder, the prompt may guide the removal of rain noise (e.g., suppressing rain line artifacts). Whereas at the deep decoder, prompt may enhance object-critical features (e.g., edges of container lock holes) to improve detection robustness. This design reduces computational complexity through a divide-and-conquer principle while achieving adaptive degradation context enhancement via fine-grained feature-prompt interactions.

The specific implementation process of the CPB Module is as follows. Firstly, generate channel attention weights $W_{i}^{c}\in R^{1\times 1\times C_{i}}$ and spatial attention weights $W_{i}^{s}\in R^{H_{i}\times W_{i}\times C_{i}}.$ to capture the key information of the input features, the calculation equation for $W_{i}^{c}$ is shown in Equation (2), and the calculation equation for $W_{i}^{s}$ is shown in Equation (3).

(2) $$W_{i}^{c}=C_{1\times 1}\left(\mathrm{ReLU}\left(C_{1\times 1}\left({\mathrm{GAP}}_{c}\left(F_{i}\right)\right)\right)\right)$$

(3) $$W_{i}^{s}=C_{7\times 7}\left([{\mathrm{GAP}}_{s}\left(F_{i}\right),{\mathrm{GMP}}_{s}\left(F_{i}\right)]\right)$$

where $C_{k\times k}$ denotes k×k convolution. ReLU represents the ReLU activation function. ${\mathrm{GAP}}_{c}$ is the global average pooling operation across the spatial dimensions. ${\mathrm{GMP}}_{s}$ is the global max pooling operation across channel attention. $\left[\begin{array}{c} \cdot \end{array}\right]$ denotes the channel-wise concatenation operation. Subsequently, the attention weights are fused with the input features, as shown in Equations (4)–(6).

(4) $$F_{i}^{w}=\left[\left(W_{i}^{c}⊕W_{i}^{s}\right)⊙F_{i},F_{i}\right]$$

(5) $$F_{i}^{s}=\sigma \left({\mathrm{DC}}_{7\times 7}\left(\mathrm{CS}(F_{i}^{w})\right)\right)$$

(6) $$F_{i}^{p}=C_{1\times 1}\left([F_{i},\mathrm{Rescale}\left(P_{i}\right)⊕F_{i}^{s}]\right)$$

where ⊙ and ⊕, respectively, represent element-wise multiplication and element-wise addition. ${\mathrm{DC}}_{k\times k}$ is the depthwise separable convolution with a stride of k×k. CS denotes the channel shuffle operation.

$F_{i}^{p}$ is split into n blocks along the channel dimension in Equation (7).

(7) $$F_{p}^{i,j}=F_{i}^{p}\left[:,:,\left(j-1\right)\frac{C_{i}}{n}:j\frac{C_{i}}{n}\right],\;j\in \left\{1,2,\ldots ,n\right\}$$

Each sub-block is input into an independent Transformer Block. Finally, the enhanced feature is obtained by concatenating the results of all sub-blocks:

(8) $$F_{i}^{g}=\left[F_{g}^{i,1},\ldots ,F_{g}^{i,j},\ldots ,F_{g}^{i,n}\right],\;j\in \left\{1,2,\ldots ,n\right\}$$

In the design of the enhancement module, we employ LDConv [26] (Linear Deformable Convolution) to generate convolution kernels of arbitrary sizes and diverse initial sampling positions. By adaptively adjusting sampling points through offsets, the convolution operation can better accommodate shape variations of the targets. This flexibility enables the model to efficiently extract critical information and enhance feature representation capabilities when processing multi-scale image data.

The approach combines hierarchical reasoning and dynamic coupled synergetic architecture construction. It can effectively deal with the multiple challenges of complex illumination, meteorological interference and geometric distortion. This approach significantly improves both semantic adaptability and structural fidelity in container image enhancement for transfer platforms.

#### 3.2.2. Multi-Scale Adaptive Frequency Object Recognition and Keypoint-Detection Method

**Figure 7:**
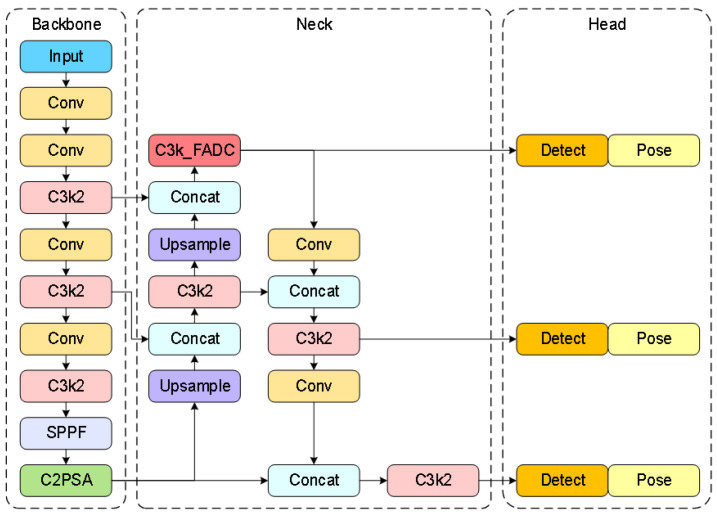
Network architecture diagram of multi-scale adaptive frequency object recognition and keypoint detection.

Traditional object-detection methods exhibit poor performance in the complex environments of container terminals, particularly in scenarios with significant scale variations of container top lockhole targets caused by perspective transformation effects from fixed cameras during container operations at transfer platforms. To address this issue, our recognition framework improves upon the conventional YOLO11 and proposes a multi-scale object-detection and keypoint-detection method, as illustrated in Figure 7.

**Figure 8:**
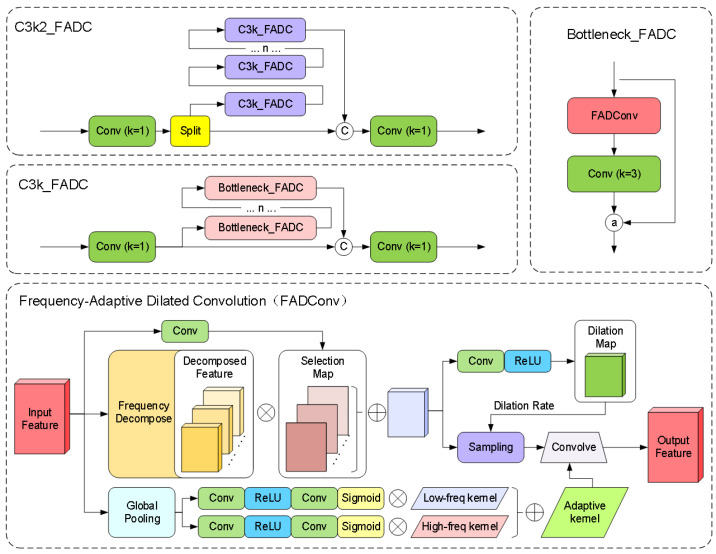
C3k2_FADC network architecture.

The original bottleneck module employs fixed-size convolution kernels, leading to insufficient information capture or excessive smoothing when processing features at different scales, thereby failing to meet the demands of diversified feature representation. Simultaneously, traditional convolution operations extract features solely in the spatial domain, exhibiting limited capacity to capture fine details under large-scale structures. Although stacking multiple layers can expand the receptive field, the fixed kernel size inherently restricts the local feature extraction capacity of single-layer convolution operations. This limitation becomes particularly pronounced in high-level semantic feature extraction or multi-scale tasks, where long-range dependency information in input data remains underutilized. To address this, we introduce Frequency-Adaptive Dilated Convolution (FADConv) into the C3k2 module for the first time, with its network architecture illustrated in Figure 8.

**Figure 9:**
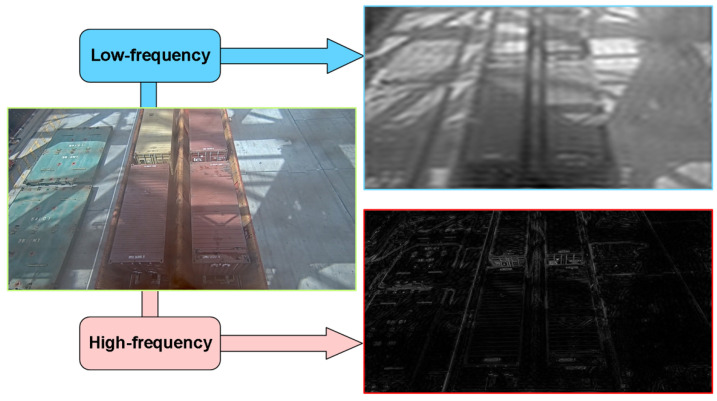
Schematic diagram of high–low frequency features in container images.

Through a frequency-adaptive mechanism, the convolutional weights are decomposed into high-frequency components and low-frequency components. As shown in Figure 9, by dynamically adjusting the convolution dilation rate and convolution kernel weight, the model can adaptively adjust its receptive field according to different local frequency characteristics, thereby enhancing its ability to capture high-frequency detail information. Additionally, the model effectively expands the receptive field of convolutional layers by balancing high- and low-frequency components. This enables the model to perform more stably when processing inputs containing both high-frequency details (e.g., textures of small-scale objects) and low-frequency structures (e.g., contours of large-scale objects), particularly in multi-scale adaptive frequency object-detection scenarios.

Specifically, this method balances Effective Bandwidth and Receptive Field through Adaptive Dilation Rate (AdaDR). The spatial dynamic dilation rate is shown in Equation (9).

(9) $$Y\left(p\right)=\sum_{i=1}^{K\times K}W_{i}X\left(p+\Delta p_{i}\times \hat{D}\left(p\right)\right)$$

where $\hat{D}\left(p\right)$ is the dynamic dilation rate at position P, predicted by a lightweight convolution. We first transform the feature map $X\in R^{H\times W}$ into the frequency domain using the Discrete Fourier Transform (DFT), it can be represented as:

(10) $$X_{F}\left(u,v\right)=\frac{1}{HW}\sum_{h=0}^{H-1}\sum_{w=0}^{W-1}X\left(h,w\right)e^{-2\pi j(uh+vw)}$$

High-Frequency Power is defined as:

(11) $$HP\left(p\right)=\sum_{\left(u,v\right)\in H_{\hat{D}\left(p\right)}^{+}}{\left|X_{F}^{\left(p,s\right)}\left(u,v\right)\right|}^{2}$$

where $H_{\hat{D}\left(p\right)}^{+}=\{\left(u,v\right)||u|>\frac{1}{2\hat{D}\left(p\right)}\;\mathrm{or}\;|v|>\frac{1}{2\hat{D}\left(p\right)}\}$ represents the high-frequency region that cannot be captured by the dilation rate $\hat{D}\left(p\right)$. Then dynamically adjust the proportion of low-frequency and high-frequency components of the convolution kernel to improve the effective bandwidth.

(12) $$\bar{W}=\frac{1}{K\times K}\sum_{i=1}^{K\times K}W_{i},\;\hat{W}=W-\bar{W}$$

where $\bar{W}$ represents the kernel-wise averaged *W*, $\hat{W}$ captures high-frequency details. Through global pooling and convolutional layers to generate channel-level dynamic weights $\lambda_{l}$ and $\lambda_{h}$, reconstructing the adaptive convolutional kernel:

(13) $$W^{\prime }=\lambda_{l}\bar{W}+\lambda_{h}\hat{W}$$

Finally, the feature spectrum is balanced through frequency band decomposition and reweighting to expand the receptive field.

(14) $$X_{b}=F^{-1}\left(M_{b}⊙X_{F}\right)$$

$M_{b}$ is the binary mask that extracts the frequency band $\left[\phi_{b},\phi_{b+1}\right)$. A spatially variant attention weight $A_{b}\in R^{H\times W}$ is applied to each frequency band $X_{b}$:

(15) $$\hat{X}\left(i,j\right)=\sum_{b=0}^{B-1}A_{b}\left(i,j\right)X_{b}\left(i,j\right)$$

This method can suppress high-frequency components in background regions, encouraging FADConv to select larger dilation rates to expand the receptive field.

**Figure 10:**
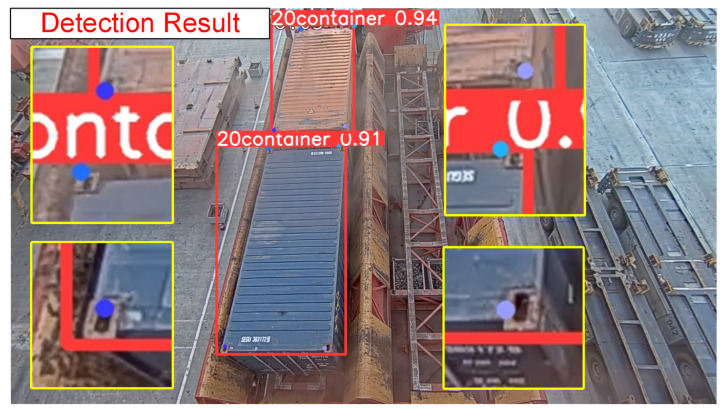
Detection results.

Based on the fundamental framework of YOLO11, combined with the multi-scale adaptive frequency object-detection method, the preprocessed images undergo precise recognition to obtain identification results for 20-foot and 40-foot general purpose containers. Simultaneously, a keypoint-detection branch is added through an inherited detection head to accurately detect the lockhole keypoints of different containers in the image and acquire their two-dimensional image coordinates. The final recognition and detection results are illustrated in Figure 10.

#### 3.2.3. Three-Dimensional Position and Pose-Measurement Method for Containers

After obtaining the container-identification results and the 2D image coordinates of the lockhole keypoints, it is necessary to calculate the position and pose of 20-foot and 40-foot general purpose containers separately. This allows determining the offset and rotation angle of the container relative to the standard position on the transfer platform. This paper proposes a three-dimensional position and pose-measurement method for containers, which consists of two parts: (1) 2D–3D Lockhole Keypoint Coordinate Conversion; (2) Container Pose Calculation.

- Step 1: 2D–3D Lockhole Keypoint Coordinate Conversion.

Assuming the coordinates of the container lockhole in the 3D world coordinate system are denoted as $P_{w}={\left[X,Y,Z,1\right]}^{T}$, and its projected coordinates in the image coordinate system are $p={\left[u,v,1\right]}^{T}$, the mapping relationship between them can be expressed as Equation (16) according to the fixed camera model.

(16) $$sp=K\left[R|t\right]P_{w}$$

Among them, *s* is the scale factor, K is the intrinsic matrix of the camera, $R\in R^{3\times 3}$ is the rotation matrix, and $t\in R^{3}$ is the translation vector. The intrinsic matrix is defined by Equation (17).

(17) $$K=\left[\begin{array}{ccc} f_{x} & 0 & c_{x}\\ 0 & f_{y} & c_{y}\\ 0 & 0 & 1 \end{array}\right]$$

In the formula, $f_{x}$, $f_{y}$ represents the focal length parameter, and $\left(c_{x},c_{y}\right)$ denotes the principal point coordinates.

Given *n* sets of 3D-2D correspondences ${\left\{P_{w}^{\left(i\right)},p^{\left(i\right)}\right\}}_{i=1}^{n}$, this work employs the EPnP algorithm (Efficient PnP) to solve the extrinsic parameters R and t. The core idea involves representing 3D points as weighted combinations of four virtual control points ${\left\{C_{j}\right\}}_{j=1}^{4}$, as shown in Equation (18).

(18) $$P_{w}^{\left(i\right)}=\sum_{j=1}^{4}\alpha_{ij}C_{j},\;\;\;\;\;\;\sum_{j=1}^{4}\alpha_{ij}=1$$

By substituting Equation (18) into the projection Equation (16), Equation (19) can be derived.

(19) $$s_{i}p^{\left(i\right)}=K\sum_{j=1}^{4}\alpha_{ij}\left({\mathrm{RC}}_{j}+t\right)$$

Through algebraic elimination of the scale factor $s_{i}$, a linear system of equations concerning control point coordinates is constructed. After solving this system using Singular Value Decomposition (SVD), the rotation matrix R and translation vector t are recovered via Orthogonal Procrustes Analysis. The specific steps are as follows:

1. Control Point Initialization: Select four non-coplanar control points, typically choosing the centroid of the 3D point set and principal component directions;
2. Weight Coefficient Calculation: Solve for $\alpha_{ij}$ using the least squares method to minimize the residual error in Equation (18);
3. Camera Coordinate System Control Point Solution: Construct an overdetermined system of equations using Equation (19) and solve it via SVD;
4. Extrinsic Parameter Recovery: Align control points in the world coordinate system with those in the camera coordinate system, minimizing registration errors as shown in Equation (20).
  (20) $$\arg\min_{R,t}\sum_{j=1}^{4}{∥C_{j}^{cam}-\left({\mathrm{RC}}_{j}^{world}+t\right)∥}^{2}$$

The containers on the transfer platform are positioned horizontally, with their upper surface lockholes also located on the same horizontal plane (Z=h). This plane constraint enhances the stability of solver computations. In this configuration, only four pairs of initial point correspondences between 2D image coordinates and 3D world coordinates are required to obtain a unique solution for the solver.

Given the 2D coordinates $p_{k}={\left[u_{k},v_{k}\right]}^{T}$ of the lockholes in the image and their height constraint $Z_{k}=h$, the 3D coordinates A are computed through back projection calculation. Equation (16) is expanded into Equation (21).

(21) $$\left\{\begin{array}{c} su_{k}=f_{x}\left(r_{11}X_{k}+r_{12}Y_{k}+r_{13}h+t_{x}\right)+c_{x}\left(r_{31}X_{k}+r_{32}Y_{k}+r_{33}h+t_{z}\right)\\ sv_{k}=f_{y}\left(r_{21}X_{k}+r_{22}Y_{k}+r_{23}h+t_{y}\right)+c_{y}\left(r_{31}X_{k}+r_{32}Y_{k}+r_{33}h+t_{z}\right)\\ s=r_{31}X_{k}+r_{32}Y_{k}+r_{33}h+t_{z} \end{array}\right.$$

By eliminating the scale factor s, we obtain the linear equation system Equation (22) concerning $X_{k}$, $Y_{k}$.

(22) $$\begin{array}{cc} \; & \left[\begin{array}{cc} f_{x}r_{11}+\left(c_{x}-u_{k}\right)r_{31} & f_{x}r_{12}+\left(c_{x}-u_{k}\right)r_{32}\\ f_{y}r_{21}+\left(c_{y}-v_{k}\right)r_{31} & f_{y}r_{22}+\left(c_{y}-v_{k}\right)r_{32} \end{array}\right]\left[\begin{array}{c} X_{k}\\ Y_{k} \end{array}\right]\\ \; & \;=\left[\begin{array}{c} u_{k}\left(r_{33}h+t_{z}\right)-f_{x}\left(r_{13}h+t_{x}\right)-c_{x}\left(r_{33}h+t_{z}\right)\\ v_{k}\left(r_{33}h+t_{z}\right)-f_{y}\left(r_{23}h+t_{y}\right)-c_{y}\left(r_{33}h+t_{z}\right) \end{array}\right] \end{array}$$

Solving Equation (22) uniquely determines the three-dimensional coordinates $\left(X_{k},Y_{k},h\right)$ of the lockhole.

- Step 2: Container Pose Calculation.

Through the acquired three-dimensional coordinates of lockholes on the container’s top surface, we analyze the container’s pose to calculate its position deviation and deflection angle on the transfer platform. Given the three-dimensional coordinates ${\left\{P_{k}=\left(X_{k},Y_{k},h\right)\right\}}_{k=1}^{4}$ of four lockholes on the container’s top surface, their geometric center coordinate $C=(C_{x},C_{y},h)$ can be calculated using the spatial point set centroid formula, as shown in Equation (23).

(23) $$C_{x}=\frac{1}{4}\sum_{k=1}^{4}X_{k},\;C_{y}=\frac{1}{4}\sum_{k=1}^{4}Y_{k}$$

For the estimation of the container’s principal direction vectors, the long-axis direction vector $v_{L}$ and short-axis direction vector $v_{W}$ are defined on the container’s top surface. By selecting long-edge container lockhole pairs, two sets of vectors are calculated as shown in Equation (24).

(24) $$v_{L1}=P_{3}-P_{1},\;v_{L2}=P_{4}-P_{2}$$

Since each container lockhole keypoint contains unique numbering, the vector direction will not be reversed due to incorrect lockhole numbering sequence, eliminating the need for direction consistency correction. A weighted average of the two vector sets yields the final long-axis direction estimation as demonstrated in Equation (25).

(25) $$v_{L}=\frac{w_{1}v_{L1}+w_{2}v_{L2}}{w_{1}+w_{2}}$$

In this scenario, the weight allocation strategy adopts equal-weight averaging, $w_{1}=w_{2}=1$.

The resolved container position and pose information are calculated with the standard position center point $C_{0}=\left(C_{x0},C_{y0},h\right)$ and standard direction vector $v_{L0}=\left(\cos\theta_{0},\sin\theta_{0},0\right)$ (preset angle: $\theta_{0}$) to obtain the positional offset and directional deviation.

The container positional deviation ΔC is shown in Equation (26).

(26) $$\Delta C_{x}=C_{x}-C_{x0},\;\Delta C_{y}=C_{y}-C_{y0}$$

By integrating dot product and cross product information, the four-quadrant arctangent function $\arctan_{2}\left(y,x\right)$ is employed to calculate the signed deviation angle as shown in Equation (27).

(27) $$\Delta \theta =\arctan_{2}\left(v_{L}\times v_{L0}\cdot e_{z},v_{L}\cdot v_{L0}\right)$$

After expansion, it is equivalent to Equation (28).

(28) $$\Delta \theta =\arctan_{2}\left(v_{L_{x}}\sin\theta_{0}-v_{L_{y}}\cos\theta_{0},v_{L_{x}}\cos\theta_{0}+v_{L_{y}}\sin\theta_{0}\right)$$

This method ensures that Δθ∈(−π,π] precisely reflects the rotational direction and magnitude of container deviation. The final solution yields the container position deviation ΔC and the rotational direction and magnitude Δθ of the container.

## 4. Experiments

### 4.1. Experimental Setup

To validate the effectiveness of the machine vision-based pose-measurement algorithm for containers on the transfer platform of dual-trolley quayside container cranes, a series of related experiments were conducted.

#### 4.1.1. Experimental Environment

The training environment parameters for these experiments are shown in Table 1.

**Figure 11:**
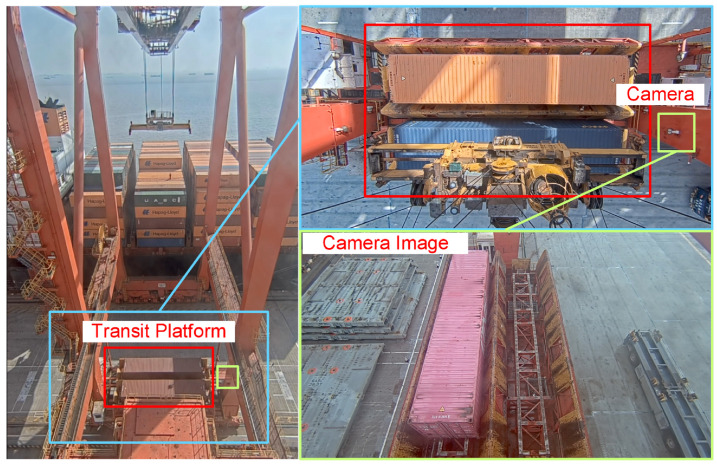
Installation position of fixed camera.

The camera used in this study is a fixed camera with adjusted and fixed shooting angles and focal lengths. This fixed camera has a pixel resolution of 1920 × 1080 and an fps of 30. The actual installation position of the fixed camera is shown in Figure 11. The red rectangle indicates the transfer platform of the dual-trolley quayside container crane, while the green rectangle marks the actual installation location of the fixed camera.

#### 4.1.2. Datasets and Evaluation Metrics

**Figure 12:**
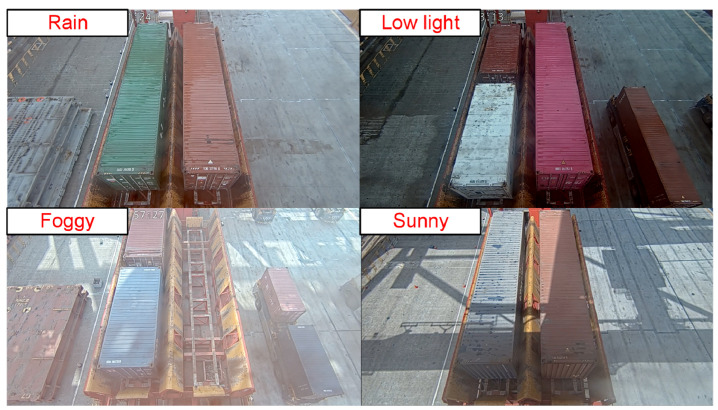
Schematic diagram of 20-foot and 40-foot general purpose container samples.

The container samples from the transfer platform were collected using fixed cameras, comprising a dataset of 4250 images. These include 20-foot general purpose containers and 40-foot general purpose containers, with samples captured under complex illumination and diverse port environmental conditions. The sample contains 827 rainy day images, 1273 sunny day images, 516 foggy day images, and 302 low light images, as illustrated in Figure 12. The richness of image samples will continue to increase with subsequent engineering deployments.

The measurement system proposed in this study primarily depends on three aspects: container-recognition and lockhole keypoint-detection accuracy, container-offset-detection accuracy, and container rotation angle-detection accuracy. To evaluate these, three core metrics are designed: detection model inference accuracy, container-offset-detection accuracy, and container rotation angle-detection accuracy.

**(a) Detection Model Inference Accuracy**

The evaluation metrics include precision, recall, and mean Average Precision (mAP). Precision refers to the ratio of true positive predictions among all positive predictions in the test data, defined as Equation (29).

(29) $$\mathrm{Precision}=\frac{\mathrm{TP}}{\mathrm{TP}+\mathrm{FP}}$$

In this context, TP (True Positive) represents the number of true positive instances, while FP (False Positive) denotes the number of false positive instances.

Recall measures the proportion of correctly identified positive class samples relative to all actual positive class samples. This is expressed in Equation (30).

(30) $$\mathrm{Recall}=\frac{\mathrm{TP}}{\mathrm{TP}+\mathrm{FN}}$$

where TP (True Positive) indicates the number of correctly predicted positive class samples, and FN (False Negative) represents the number of samples that are actually positive but were erroneously predicted as negative.

mAP (mean Average Precision) is the average of the Average Precision (AP) values computed for each detection category. This experimental section employs two evaluation metrics: mAP@0.5 and mAP@0.5:0.95. Specifically, mAP@0.5 is defined as the mean average precision calculated with an Intersection over Union (IoU) threshold of 0.5, where a detection is considered correct if the IoU between the predicted bounding box and the ground truth bounding box is ≥0.5. To comprehensively evaluate model performance under varying localization accuracy requirements, particularly focusing on detection capability at high IoU thresholds, this study additionally calculates the average mAP across 10 distinct IoU thresholds ranging from 0.5 to 0.95 with a step size of 0.05.

**(b) The detection accuracy of container horizontal deviation**

In the experimental evaluation, to quantify the translation-detection accuracy of containers along coordinate axes within the planar coordinate system, this study conducts independent error analyses on the offset deviations along the *X*-axis and *Y*-axis within the horizontal plane. For the offset deviations $\Delta C_{x}$ in the *X*-axis direction and $\Delta C_{y}$ in the *Y*-axis direction, the evaluation metrics are defined as the deviations from manually measured ground truth values $\Delta C_{x0}$ and $\Delta C_{y0}$.

Single-axis Mean Absolute Deviation (MAD): To characterize systematic errors in single-axis offset deviations, this metric calculates the mean absolute deviation across all samples, as shown in Equation (31).

(31) $${\mathrm{MAD}}_{X}=\frac{1}{N}\sum_{i=1}^{N}\left|\Delta C_{x}^{\left(i\right)}-\Delta C_{x0}^{\left(i\right)}\right|,\;{\mathrm{MAD}}_{Y}=\frac{1}{N}\sum_{i=1}^{N}\left|\Delta C_{y}^{\left(i\right)}-\Delta C_{y0}^{\left(i\right)}\right|$$

Mean Absolute Error in the Plane (MAE-P): To further evaluate the overall translation accuracy within the horizontal plane (*XY* plane), we define the mean value of two-dimensional projection absolute errors, as shown in Equation (32).

(32) $$\mathrm{MAE}-P=\frac{1}{N}\sum_{i=1}^{N}\sqrt{{\left(\Delta C_{x}^{\left(i\right)}-\Delta C_{x0}^{\left(i\right)}\right)}^{2}+{\left(\Delta C_{y}^{\left(i\right)}-\Delta C_{y0}^{\left(i\right)}\right)}^{2}}$$

**(c) The detection accuracy of container rotational deviation**

In experimental evaluations, for the calculated container rotation angle Δθ and manually measured rotation angle $\Delta \theta_{0}$, we employ the Mean Absolute Error of Rotation Angle (MAE-θ) to establish an angular error evaluation system, shown in Equation (33).

(33) $$\mathrm{MAE}-\theta =\frac{1}{N}\sum_{i=1}^{N}\left|\Delta \theta^{\left(i\right)}-\Delta \theta_{0}^{\left(i\right)}\right|$$

### 4.2. Experimental Results

#### 4.2.1. Model Accuracy Testing

**Figure 13:**
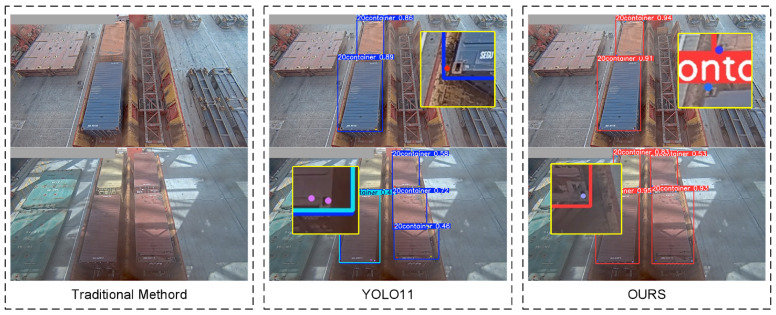
Algorithm comparison results.

To validate the effectiveness of the adaptive enhanced image feature preprocessing method and multi-scale adaptive frequency object detection with keypoint detection for container recognition and lockhole detection on container roofs, comparative experiments were conducted using traditional algorithms, the original YOLO11 algorithm, and our improved algorithm. The visual comparison results are shown in Figure 13.

**Figure 14:**
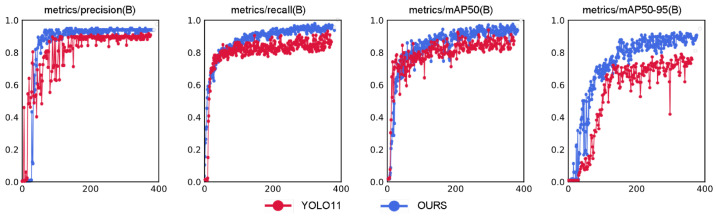
Training results comparison between our method and YOLO11 algorithm.

The comparison of training results between our proposed adaptive enhanced image feature preprocessing method and the multis-cale adaptive frequency object-detection algorithm versus the original YOLO11 network is illustrated in Figure 14. The blue line represents our method, while the red line denotes the original YOLO11 network. The horizontal axis indicates the number of training epochs.

From the training comparison graph, we observe that both methods exhibit rapid increases in precision curves at similar rates during the initial training phase. However, our method demonstrates significantly smaller oscillation amplitudes compared to the YOLO11 model, reflecting stronger robustness against noisy samples in the optimization process. Concurrently, our method achieves faster convergence speed than YOLO11, with higher accuracy and reduced oscillations between 200–400 epochs. In the recall curve, our model shows superior capability in recalling true targets compared to YOLO11, with smoother convergence and smaller late-stage oscillations, indicating enhanced localization ability for occluded and multi-scale targets. The mAP50 curve reveals that our method stabilizes after 200 epochs while significantly outperforming YOLO11. The mAP50-95 curve further confirms the consistent superiority of our proposed model. These results demonstrate substantial improvements in target-recognition accuracy, localization precision, and overall algorithm performance. The experimental results are summarized in Table 2.

As can be seen from the table, our method demonstrates significant advantages over traditional approaches in terms of precision (P), recall (R), mean Average Precision (mAP@0.5), and mAP@0.5:0.95. The reason for this construction is mainly due to the fact that the traditional SIFT algorithm is based on localized features and is sensitive to changes in lighting, viewing angle, etc., which can easily lead to matching failures in this scenario. Our approach is also significantly improved compared to YOLO11. Specifically, the precision is improved by 3.7% and recall by 4.2% compared to YOLO11, indicating dual optimization of the model’s capability in handling large-scale variation target classification and target-localization completeness under complex illumination and challenging port environments. Concurrently, the mAP@0.5 shows a 4.7% enhancement and mAP@0.5:0.95 an 8.7% improvement, reflecting substantial progress in basic detection capabilities and enhanced multi-scale target-recognition and -localization abilities. Compared with the multi-scale target-detection algorithm HRNet, all evaluation metrics are improved but not significantly. However, the larger number of parameters in HRNet requires more computational resources, and the lighter YOLO algorithm is more suitable for deployment in port operation environments where computational resources are limited. These results validate the effectiveness and superior recognition accuracy of the proposed vision-based container-identification and lockhole keypoint-localization methodology. At the same time, we noticed a complete loss of distinguishable structure of the lock hole in the image under extreme low light and extreme high light reflections. There are some limitations of our algorithm in that case.

#### 4.2.2. The Detection Accuracy of Container Horizontal Deviation

**Figure 15:**
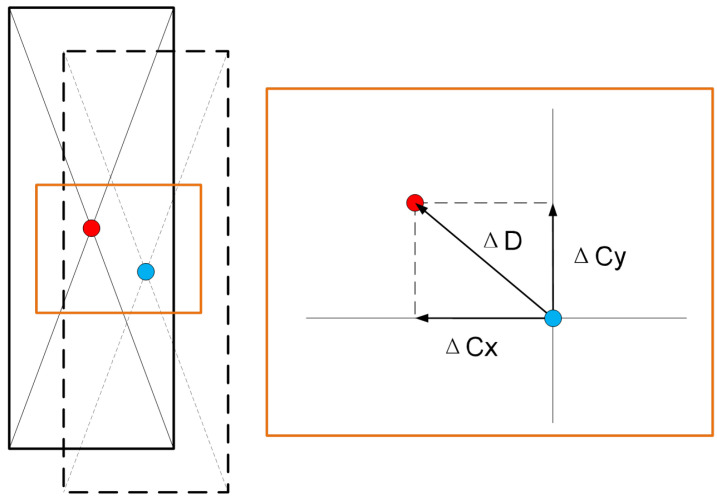
Schematic diagram of horizontal deviation.

To verify the precision of container positioning calculation, this paper compares the proposed three-dimensional position-measurement method with traditional manual measurements through a complete quayside container crane operation cycle. Independent error analyses are conducted for horizontal deviations along both the *X*-axis and *Y*-axis directions. Figure 15 is the schematic diagram of horizontal deviation.

The method is scientifically evaluated through Mean Absolute Deviation (MAD) for single-axis analysis and Mean Absolute Error in the Plane (MAE-P), providing comprehensive assessment from both individual axes and integrated planar perspectives.

The comparison results between the method proposed in this paper and manual operations in Table 3 demonstrate that the proposed method achieves a precision error of 0.012 m on the *x*-axis under the Mean Absolute Deviation (MAD), outperforming the manual operation’s precision error of 0.013 m. However, its *y*-axis precision error of 0.018 m is slightly higher than the manual operation’s 0.016 m. In terms of the Mean Absolute Error in the Plane (MAE-P), the proposed method achieves a precision error of 0.024 m, reaching the level of manual operation (0.023 m). Additionally, the proposed method reduces the average operation time by 0.68 s compared to manual operations.

#### 4.2.3. The Detection Accuracy of Container Rotational Deviation

**Figure 16:**
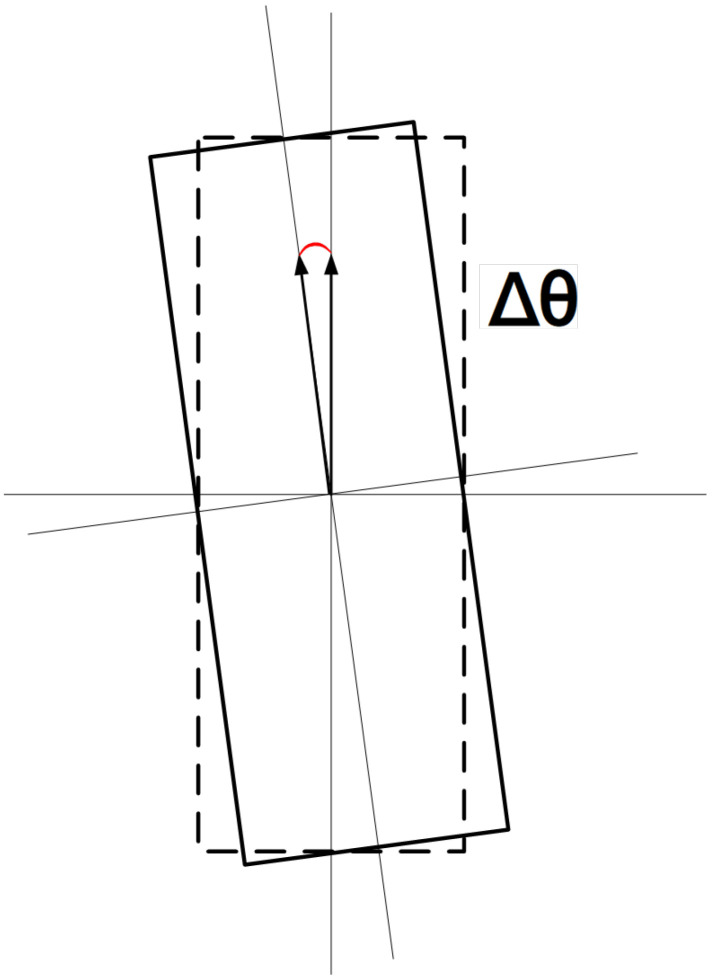
Schematic diagram of deflection angles.

To verify the accuracy of container deflection angle calculation, this paper conducted comparative experiments between the proposed three-dimensional container pose-measurement method and traditional manual measurement results through a quayside crane operation cycle. The comparison was established using the Mean Absolute Error of Rotation Angle (MAE-θ) as the angular error evaluation metric. Figure 16 is the schematic diagram of deflection angles.

From the comparative experimental results between the proposed method and manual operations in Table 4, it can be observed that the proposed method achieves a MAE-θ of 0.11°, outperforming the manual operation’s average error of 0.15°. Additionally, it demonstrates superior average operation time by 1.15 s compared to manual operations. The smaller MAE-θ indicates higher alignment precision, while manual operations tend to rely on spreader guide plates sliding into containers. Therefore, the proposed method not only saves operation time but also potentially reduces wear between spreader guide plates and containers to some extent.

## 5. Conclusions

As the core equipment for efficient container handling operations, the dual-trolley quayside container crane relies on its critical connecting component—the container-transfer platform—to perform essential container-transfer functions between the main trolley and gantry trolley. To address the technical challenge of container pose measurement on the transfer platform, this study proposes a high-precision vision-based measurement system. The hardware system integrates fixed cameras with edge computing modules. An adaptive image-enhancement preprocessing algorithm enhances image feature robustness under complex illumination conditions. A multi-scale adaptive frequency object-detection framework is developed based on YOLO11, achieving significant improvement in multi-scale lockhole keypoint-detection accuracy through dynamic balance of high–low frequency features and adjustable deformable convolution kernels in perspective-distortion scenarios. An improved EPnP optimization algorithm incorporating lockhole coplanarity constraints establishes a 2D–3D coordinate transformation model, reducing pose solution errors to millimeter-level positional accuracy and sub-degree angular precision. Experimental validation confirms the effectiveness of this algorithm for container pose measurement on dual-trolley quayside container crane-transfer platforms. This method provides automated ports with an efficient and cost-effective solution for container pose measurement, effectively reducing spreader adjustment time and mechanical wear while enhancing operational efficiency and safety of dual-trolley quayside container cranes. The demonstrated practicality and effectiveness highlight its engineering application value.

## Figures and Tables

**Table 1:** Training environment parameters for this experiment.

Hardware/Software	Configuration Parameters
CPU	Intel(R) Xeon(R) CPU E5-2690
GPU	NVIDIA GeForce RTX 3090
Memory	64 GB
Operating System	Ubuntu 20.04
Programming Language	Python = 3.10
Deep Learning Framework	PyTorch = 2.0

**Table 2:** Algorithm comparison experimental results.

Methods	P (%)	R (%)	mAP@0.5 (%)	mAP@0.5:0.95 (%)
Traditional	24.1	12.6	/	/
HRNet	90.3	88.6	92.1	88.1
YOLO11	89.7	88.3	90.4	80.9
OURS	93.4	92.5	95.1	89.6

**Table 3:** Experimental results of the detection accuracy of container horizontal deviation.

Methods	Mean Absolute Deviation, MAD		MAD-P (m)	Average Operation Time (s)
	${\mathrm{MAD}}_{X}$ (m)	${\mathrm{MAD}}_{X}$ (m)		
Manual operation	0.013	0.016	0.023	9.36
Ours	0.012	0.018	0.024	8.68

**Table 4:** Experimental results of container deflection angle-detection accuracy.

Methods	MAE-θ (°)	Average Operation Time (s)
Manual Operation	0.15	9.86
OURS	0.11	8.71

## Data Availability

Data are contained within the article.
